# The factualization of ‘I suppose’ in American English: a corpus based study of the subjectification of epistemic predicates toward factuality

**DOI:** 10.1186/s40064-016-3438-0

**Published:** 2016-10-12

**Authors:** Vittorio Tantucci

**Affiliations:** Department of Linguistics and English Language, Lancaster University, C87, County South, Bailrigg, Lancaster, LA1 1YW UK

## Abstract

This work provides a case study centered on the cognitive phenomenon of *factualization*, viz. “the SP/W’s increasing certainty about the realization of an event or situation” (cf. Tantucci 2014, 2015a, b, 2016b). Factualization corresponds to a cognitive-control mechanism (i.e. Kan et al. 2013) specifically occurring in the epistemic domain. It instantiates both in online language production and throughout the diachronic reanalysis of a construction (i.e. grammaticalization, semasiological change or constructionalization, cf. Traugott and Dasher 2002; Traugott and Trousdale 2013). The case presented here focuses on the diachronic change of the epistemic construction *I suppose* in British English. It will be shown that *I suppose* developed through time an increasingly factual usage out of an original meaning conveying weak epistemicity. Qualitative and quantitative data from the Corpus of Historical American English will support the general claim that—to varying degrees—epistemic predicates diachronically tend to develop new polysemies encoding a Speaker/writer’s (henceforth SP/W) “subjectified form of certainty” towards a proposition P (cf. Tantucci 2015a: 371).

## Background

This work provides a case study centered on the cognitive phenomenon of *factualization*, viz. “the SP/W’s increasing certainty about the realization of an event or situation” (cf. Tantucci 2014, 2015a, b, 2016b). Factualization corresponds to a cognitive-control mechanism (i.e. Kan et al. 2013) specifically occurring in the epistemic domain. It instantiates both in online language production and throughout the diachronic reanalysis of a construction (i.e. grammaticalization, semasiological change or constructionalization, cf. Traugott and Dasher 2002; Traugott and Trousdale 2013).

The case presented here focuses on the diachronic change of the epistemic construction *I suppose* in BE (British English). It will be shown that *I suppose* developed through time an increasingly factual usage out of an original meaning conveying weak epistemicity. Qualitative and quantitative data from the Corpus of Historical American English (COHA,^1^ cf. Davies 2010) will support the general claim that—to varying degrees—epistemic predicates diachronically tend to develop new polysemies encoding a Speaker/writer’s (henceforth SP/W) “subjectified form of certainty” towards a proposition P (cf. Tantucci 2015a: 371). In cognitive psychology, recent studies on perceptual/linguistic cognitive control and ‘conflict monitoring theory’ (cf. Norman and Shallice 1986; Desimone and Duncan 1995; Botvinick et al. 2001; Miller and Cohen 2001; Schlaghecken and Martini 2012), have shown that “experiencing [perceptual or linguistic] ambiguity appears sufficient to yield conflict adaptation” (Kan et al. 2013, p. 647). Accordingly, this study will provide evidence to show that epistemic uncertainty is itself a form of cognitive conflict between two propositions: *P is true* vs. *P is false*. In this sense, synchronic and diachronic phenomena of factualization are to be intended as a general embodied impulse to resolve epistemic conflicts in favor either of the former (*P is true*) or the latter (*P is false*). This paper will provide diachronic evidence to support confirm that cognitive control mechanisms can be operationally observed to occur in the epistemic domain. It is organized as follows: in “On factuality” section I provide a brief overview about the notion of factuality where a special emphasis is given to Narrog (2002, 2005a, b, 2009, 2012) and Tantucci (2015a) approaches. In “On factualization” section I discuss the notion of fatualization as a form of semasiological subjectification. In “The factualization of *I suppose* in American English” section the main case-study of this paper is given, as I provide quantitative and qualitative data about the factualization of *I suppose* from the COHA across the nineteenth and the twentieth century.

### On factuality

Factuality in the literature is alternatively labeled as ‘realis’ (e.g., Mithun 1999; Palmer 2001), ‘factivity’, ‘reality’, ‘actuality’ (e.g., Kiparsky and Kiparsky 1971; Chung and Timberlake 1985; Papafragou 2000), or ‘validity’ (Kiefer 1987; Dietrich 1992). It broadly refers to the pragmatic, semantic or grammatical encoding of a proposition that is communicated as a ‘fact’, or in other words, as an event/situation posited as ‘real’, in opposition to what is unreal, hypothetical or possible.

In Narrog (2002, 2005a, b, 2009, 2012) factuality is counterposed to modality in that he defines the latter as the domain marking the non-factuality or ‘undetermined-factuality’ of an event. With this premise, modally unmarked assertions are generally employed to posit a situation/event as a fact (Narrog 2005b: 187):

(1): mary is at home now.
(2): mary **may** be at home now.

In Narrog’s account, the main semantic function of the epistemic modal *may* in (2) is to suspend the factual meaning conveyed in (1). Along a similar line of thought, in Tantucci (2015a) it is pointed out that factuality in language is entailed by SP/W’s marked certainty about the state of affairs of a situation. In this sense, factual statements can be pragmatically paraphrased as *As (I am sure that) P is true, P*. This is tested in (1a) below, which is logically inconsistent, in comparison with in (2a), which is perfectly acceptable:

(1): (a) *Mary is at home now, **(although) I am not sure**.
(2): (a) Mary may be at home now, **(although) I’m not sure**.

(2a) above—is semantically open to challenge as it suspends the factual status of the utterance. Quite differently, the factual assertion (1a) entails SP/W’s subjective certainty about the actualization of the event *Mary being at home* in the real world. It follows from this that “an assertion is pragmatically and logically factual as long as it is not marked by constructions encoding epistemic uncertainty” (cf. Tantucci 2015a: 374). On the other hand, modally marked propositions are logically consistent with constructions expressing doubtfulness or hesitancy on behalf of SP/W.

### On factualization

The process of factualization can be observed diachronically or during online speech production. It corresponds to the SP/W’s increasing certainty about the realization of an event or situation (cf. Tantucci 2014, 2015a, b, 2016b). Diachronically, factualization occurs in the form of ‘subjectification’ (Traugott 1989, 1995, 2003, 2010, 2012; Traugott and Dasher 2002; Langacker 2008, 2009). The latter notion is generally addressed semasiologically, viz. by focusing on a form–meaning pair L (lexeme or construction) and the changes that the meaning M of L undergo through time (cf. Geeraerts 1997). Simply put, “subjectification is the semasiological process whereby linguistic expressions acquire subjective meaning. In particular, it refers to the tendency whereby lexical material tend[s] to become increasingly based in the SP/W’s subjective belief state or attitude to what is being said and how it is being said. (Traugott 2003: 25; see also 1989: 35, 1995: 47)”.

The literature on subjectification in the last 15 years is extremely vast and diverse. A famous example of epistemic subjectification is first given in Sweetser (1990: 52) who proposes that the epistemic domain is to be understood in terms of a metaphorical mapping from the socio-physical world of obligation (the ‘root’/deontic domain) to the world of reasoning (the epistemic domain):

(3): (a) You **must** be at home by ten. (Mom said so.) [deontic]
  (b) You **must** have been home last night. [epistemic]

(Sweetser 1990, p. 61)

To explain, *must* in (3b) above is comparatively more subjectified that it is in (3a) as it encodes SP/W’s personal belief towards a proposition P. As put by Sweetser, in (3a) “the direct force of mom’s authority compels you to come home by ten” (1990, p. 61) with SP/W exerting external control over the AD/H: s/he tries to affect directly the state of affairs of AD/R’s actions. Quite differently, in (3b) *must* is comparatively more subjectified as SP/W exerts a form of epistemic control over a proposition P. In this latter case, SP/W is making a subjective attempt to find some certainty about a proposition P (see Nuyts 2001; Tantucci 2013, 2016a on the intersections between subjectivity and epistemic modality). Correspondingly, Traugott (1989: 43) argues that some modals in English not only show a diachronic shift from non-epistemic to epistemic, but also from relatively ‘weak’ to ‘strong’ epistemicity.

In line with this idea, Tantucci (2015a) provides a synchronic collostructional study (cf. Stefanowitsch and Gries 2003) about the epistemic polysemy of the BE predicates *I think, I believe* and *I reckon* and a diachronic corpus-based survey about the process of factualization of *Io penso* ‘I think’ in Italian. What emerges from the results of both studies is that epistemic predicates encoding different levels of (un-)certainty all seem to progressively develop new polysemies expressing a subjectified form of certainty. In other words, it shown statistically that epistemic predicates expressing different levels of conjecture or guess diachronically all tend to be increasingly employed in contexts of factuality.

Factualization theory is grounded in Langacker’s ‘epistemic control cycle’ model (cf. Langacker 2008, 2009). The latter essentially provides a constructional taxonomy of different stages of confidence according to which SP/W considers P as a fact. What crucially emerges from the data presented in Tantucci (2015a) is that predicates originally expressing a comparatively weaker form ‘epistemic inclination’ towards the truthfulness of P, show a general diachronic tendency to expressing ‘epistemic result’, viz. a new subjectified form of certainty upon the factuality of P.

Examples (4) and (5) below are representative respectively of an inclinational usage of *Io penso* ‘I think’ in the time span 1861–1900 and one of epistemic result in the last period 1968–2001 from the diachronic corpus of written Italian (cf. Onelli et al. 2006):

(4): Nel tempo in cui l’ imperatore Enrico soggiogò la Sicilia, era nella Chiesa di Palermo un decano, di nazione, secondo ch’**io penso** tedesco.
  ‘At the time when the emperor Enrico subjugated Sicily, in the Church of Palermo there was a dean, his nationality was, I think, German.’

(diaCORIS – Saggistica – Miti, Leggende e superst. del Medio Evo – Graf Arturo 1892)

(5): francamente **penso** che la democrazia deve ora fare il massimo sforzo revisionistico ed evolutivo (a sinistra)
  ‘Frankly, I think democracy has now to make a greatest revisionist and evolutionary e ort (to the left)…’.

(diaCORIS – Miscellanea – Una scelta di vita – Amendola Giorgio 1976)

In (4) *Io penso* expresses a positive attitude towards the factuality of P, yet not absolute certainty. This can be easily tested by adding an inclinational mitigator such as *anche se non ne sono sicuro* ‘although I’m not sure’. Quite differently (5) corresponds to a statement of epistemic-result, expressing a subjectified form of factuality. In fact, the indicative form (conveying factuality) is here employed instead of the expected subjunctive one (the grammatical mood expressing irreality in Italian) after mental state predicates or ‘verba dicendi’. Moreover, different from (4) above, now the addition of an inclinational element like *anche se non ne sono sicuro* ‘although I’m not sure’ will now lead to logical inconsistency:

(4): (a) Nel tempo in cui l’ imperatore Enrico soggiogò la Sicilia, era nella Chiesa di Palermo un decano, di nazione, secondo ch’ **io penso** tedesco, *anche se non ne sono sicuro*.
  ‘At the time when the emperor Enrico subjugated Sicily, in the Church of Palermo there was a dean, his nationality was, I think, German, although I am not sure.’
(5): (a) *Francamente **penso** che la democrazia deve ora fare il massimo sforzo revisionistico ed evolutivo (a sinistra), *anche se non ne sono sicuro*.
  ‘Honestly, I think democracy now has to make a greatest revisionist and evolutionary e ort (to the left), although I’m not sure.’

Despite the recent findings on diachronic and synchronic cases factualization (i.e. Traugott 1989; Tantucci 2015a, 2016b), me must note that elsewhere it is proposed that epistemic adverbials do show a tendency to acquire a more ‘discourse’ function, which is argued to convey a lesser degree of factuality (cf. Capone 2001). Similarly, Capone also suggests that verbs of knowledge seem to become epistemically weaker (Capone 2000) whereby clitics appear to compensate this trend (Capone 2013). These points might suggest that more cases of factualization phenomena need to be empirically observed before we can draw general conclusions about factualization as a general tendency of change. Important to note is also that clines of change of the so-called ‘weaking kind’ are observed in qualificational shift from epistemicity to evidentiality (cf. Nuyts 2012), viz. ones that are characterized by a shift from evaluational to presentative illocutionary force (cf. Tantucci 2016a). However, it is still under debate wether along a merely epistemic-modal cline of change phenomena of epistemic weakening (or de-factualization) have occurred at all. This work aims at extending the application of factualization theory. It will be emphasized that diachronic factualization constitutes a cognitive phenomenon which can be observed cross-linguistically. To achieve this, the rest of this paper provides a case-study about the diachronic factualization of *I suppose* across the nineteenth and the twentieth century in American English.

## The factualization of *I suppose* in American English

Mental verbs carrying an epistemic meaning, such as *I think, I believe* or *I reckon* are polysemous: they originally indicate a specific mental activity, i.e. *the act of thinking* without an epistemic stance being conveyed. Through time, they progressively acquire a more central argumentative use: *I think P as P is my personal opinion.*

 In the latter case they can express whether SP/W is inclined to believe that P is true, whether SP/W has reached the conclusion that P is true or they can ultimately express whether SP/W is subjectively sure about the truthfulness of P. Concerning this point, Simon-Vandenbergen (1996: 405–406) points out that *I think* in different contexts can express lack of commitment as well as certainty. Holmes (1990: 187) also distinguishes between tentative and deliberative usages of *I think*, the former profiling a limited commitment to the truth, the latter conveying confidence and certainty (cf. also Holmes 1984: 354). In a similar fashion, Traugott (1995: 38) argues that *I think* developed a more subjectified meaning conveying a speaker’s epistemic attitude. Nuyts (2001: 113) also considers that the verb *to think* can express either epistemic possibility or certainty. Tantucci (2015a) observes that epistemic predicates show a tendency to acquire new factual polysemies through time, viz. from epistemic inclination (expressing guess or conjecture) to epistemic result (conveying SP/W’s certainty). Interestingly while evidence suggests that factualization occurs as a widespread and unidirectional phenomenon, there is no data in the literature that might suggest cases of de-factualization, viz. a process of semantic change of verbs of certainty towards uncertainty.

Similar to *I think, I believe* or *I reckon*, the predicate *I suppose* is polysemous, as it may either express epistemic inclination (viz. the SP/W’s positive intention to consider P as a fact) or epistemic result (viz. SP/W’s subjectified certainty about P). Consider the following synchronic examples:

(6): well, he started playing footsie–footsie with me […]. **I suppose** he **might** have had cramp or something.

BNC

(7): I **suppose** I **absolutely** must marry.

COCA

In (6) above the usage of *I suppos*e encodes SP/W’s epistemic inclination as SP/W gives a tentative explanation for someone’s behavior. The inclinational force of the utterance in (6) is constructionally made explicit through the employment of the modal *might,* which is adopted to markedly suspend the factuality of P (cf. Narrog 2002, 2005a, b, 2009, 2012; Tantucci 2015a). On the other hand, *I suppose* in (7) appears in a statement expressing SP/W’s subjective confidence about the truthfulness of P. This is due to the felicitous co-occurrence of *I suppose* with the predicate *absolutely,* the latter inherently expressing epistemic result. In fact, while SP/W’s statement in (6) cannot be presupposed as a fact in a subsequent proposition, *I suppose* in (7) can be felicitously referred back as a factual statement:

(6): (a) Well, he started playing footsie–footsie with me […]. **I suppose** he **might** have had cramp or something. * *His cramp was due to P*.
(7): (b) I **suppose** I **absolutely** must marry. *The reason I must marry is that P*.

To better explain, it is generally agreed that presuppositions semantically encode factuality. They correspond to an implicit assumption about the world or background belief relating to an utterance whose truth is taken for granted in discourse (Stalnaker 1974, 1999, 2002; Tantucci 2016b). A presupposition refers a proposition Q the factuality of which is taken for granted by the producer of an utterance and which must be known and taken account of for the utterance to make sense to an interpreter (cf. Cruse 2006: 138; Fetzer 2011: 32). Presuppositions instantiate through specific constructions, which in the literature are generally referred to as **presupposition triggers PT** (cf. Stalnaker 1974, 1999, 2002; Delogu 2009; Huang 2011; Fetzer 2011). PT formally correspond to constructional instantiations of a presupposed element Q: i.e. temporal clauses, cleft sentences, counterfactual conditionals and other constructions.

In the cases of (6) and (7) above, the cleft-sentences *his cramp was due to P* and *the reason I must marry is that P* both presuppose Q as a fact, respectively: *he had cramps* and *I must marry*. However, while in the case of (7) a presupposition is logically allowed, in the case of (6) the factual meaning expressed through the presupposition *his cramp was due to* P is not epistemically consistent with the previous inclinational construction *I suppose he might have had cramp*. As a result of this, it can be concluded that the degree of subjectified factuality of an epistemic predicate (i.e. *I suppose, I think* and so on) can empirically tested by looking at whether P may be subsequently presupposed as a fact.^2^

### The factualization of *I suppose* in American English: a method of enquiry

SP/W’s epistemic stance is often communicated through the intersection of predicates expressing different degrees of beliefs/certainty together with additional surrounding elements, i.e. epistemic modals or adverbials. In the case of (6) and (7) above, the illocutionary force expressed by SP/W shifts from inclination to result precisely due to the co-occurence of surrounding items such as *might* or *absolutely.*

What is of interest for the present analysis is to assess the degree of co-occurrence of an epistemic predicate of weak certainty (i.e. *I suppose*, *I reckon*) with additional elements which may contribute to conveying a subjective ‘factualized’ meaning. In other words, this work aims at providing additional evidence to show that epistemic predicates tend to become increasingly ‘factual’ over time, in the sense that they tend to occur more and more frequently in contexts where SP/W expresses a subjectified form of certainty. To demonstrate this on a quantitative level, I consulted the diachronic corpus of American English (COHA) and selected the 100 most frequent adverbial co-occurrences with *I suppose* within a 1L-4R word-span (cf. Capone 2001 on modal adverbs and discourse). Among those, I then restricted my analysis to all the adverbials conveying either an inclinational (i.e. *maybe, possibly*) or a result epistemic meaning (i.e. *surely, absolutely*). The identification of inclinational vs. result adverbials was based on the test provided in (6a–7a). See (8–9) below:

(8): A: I understand you’re looking for a job.
  B: Yes, I am. **I suppose** I’m **really** looking for a home.

COHA Enchanted Cottage 1945

(8): (a) B: Yes, I am. **I suppose** I’m **really** looking for a home. *The reason why I am looking for a home is* P.
(9): I **suppose maybe** you think something might have happened to me or something.

COHA The Real Dope 1919

(9): (a) I **suppose maybe** you think something might have happened to me or something. * *The reason why you think something might have happened is* P.

Similar to the case in (6–7), even in (8–9) above it is possible to disentangle an inclinational meaning from a result one. In fact, while in (8a) the complement clause of *I suppose* can be subsequently presupposed as a fact, in the case of (9a) the inclinational meaning of *I suppose* co-occurring with *maybe* cannot be subsequently presupposed as a factual statement.

The raw frequency from this dataset is given in the Tables 1, 2 encompassing a period of 160 years below (from 1810 to 1960):

**Table 1:** Raw frequency of result adverbials from the COHA (1810–1960)

	1810	1820	1830	1840	1850	1860	1870	1880	1890	1990	1910	1920	1930	1940	1950	1960
*really*				1	3	2	3	6	8	1	12	14	15	12	18	13
*hardly*		1	2	1	4	2	7	3	3	3	3		1			
*actually*					1		1			1	2	3	4	3	4	4
*certainly*					1	1	2	4		1	1					1
*indeed*						2	3	1	2					1		
*sure*						1						1		1	1	
*exactly*					1	1		1		2	1	2	2		1	2

**Table 2:** Raw frequency of inclination adverbials from the COHA (1810–1960)

	1810	1820	1830	1840	1850	1860	1870	1880	1890	1990	1910	1920	1930	1940	1950	1960
*maybe*					1					1	2	5	1	1	1	4
*perhaps*	1	1		1	2	3	1	2	2	3	1	3	1		2	2
*probably*				1		2				1	1	1			2	2
*likely*			1			4				1	1	2				

**Fig. 1:**
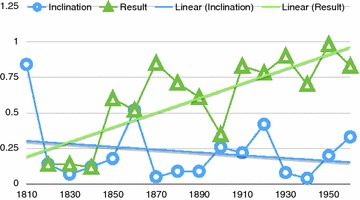
NF of result and inclinational adverbials co-occurring with *I suppose* from 1810 to 1960

The normalized frequency per each decade of respectively result and inclinational adverbials co-occurring with *I suppose* is visually given in the Fig. 1.

As the two straight trend lines^3^ encompassing the whole period suggest, result adverbials show an increasing tendency to collocate with *I suppose*, while the normalized co-occurrence with inclinational adverbials is slightly decreasing.

What crucially emerges from the data above is that while result usages of a predicate of weak epistemicity such as *I suppose* are diachronically increasing, on the other hand adverbs expressing epistemic inclination do not show the same tendency. Even more importantly, the difference between result and inclinational usages of *I suppose* during the first 8 decades is significantly lower in comparison with the period running from 1890 till the end of 1960 (Fisher exact test, *p* < 0.0005).

Given these points, we may conclude that *I suppose* underwent a process of factualization across the nineteenth and the twentieth century as it shows an increasing tendency to appear in contexts where SP/W idiomatically expresses a subjectified form of certainty. This evidence has been provided to support the diachronic unidirectional hypothesis of factualization.

## Conclusion

Evidence suggests that factualization is a universal phenomenon, which can both be tested diachronically or during online language production. Focusing on the former, the present work supported the idea that factualization instantiates semasiologically as a form of subjectification. It complemented the hypothesis proposed in Tantucci (2015a) about the unidirectional tendency of epistemic predicates to develop new factual polysemies and to be increasingly employed in contexts where SP/W expresses a subjectified form of certainty. The quantitative and qualitative analysis of this study is centered on the semantic change of the predicate of weak epistemicity *I suppose* across a time span of 160 years from the Corpus of Historical American English.

## Footnotes

1 Project website: http://corpus.byu.edu/coha/. Last accessed 2-10-15.

2 As suggested by a reveiwer, it may be argued that an expression like *I suppose* may need not be considered as a construct conveying uncertainty but rather a mere mental activity of a general type. As a result, a proposition such as *I suppose John is at home, I saw his car parked here* would simply mean *I know by deduction that he is at home.* On the other hand, the meaning of *I suppose John is at home* cannot be merely restricted to the ‘mental act of supposing’. From a pragmatic angle, the statement directly triggers a conversational—or rather conventional—implicature that directly impinges on SP/W’s epistemic stance: *I am (inclined) to conclude that* P. By uttering so, SP/W is not necessarily conveying that *I know that* P, as s/he can subsequently defeat P by suspending its factuality: i.e. *but who knows?*, *though I am not sure*. The usage is to be considered as ‘factualized’ in constructs that include adverbials of results, entailing absolute factuality, such as the afore mentioned *absolutely*.

3 In statistics, trend-line calculation (which can be automatically obtained in most of graph-editing softwares available) is an approach for modelling the average relationship between a scalar dependent variable y and one or more explanatory variables denoted x.
